# Supplementary material for: The Effect of the Supplementation of a Diet Low in Calcium and Phosphorus with Either Sheep Milk or Cow Milk on the Physical and Mechanical Characteristics of Bone using A Rat Model
Source: Foods. 2020 Aug 7;9(8):1070. doi: 10.3390/foods9081070 (PMC7466322; doi:10.3390/foods9081070)
Supplement: Supplementary file 1 [file foods-09-01070-s001.pdf]

Table 1. Sheep milk, cow milk, modified-AIN-93M diet, and Low Ca/P modified-AIN-93M diet mineral compositions as determined by ICP-MS [%]

Diet component	Basal		Milk	
Element	Modified-AIN-93M [μg/kg]	Low Ca/P modified-AIN-93M [μg/kg]	Sheep [μg/kg]	Cow [μg/kg]
Al *	8.32	9.68	1.72	N/A
Ba	N/A	N/A	741	167
Ca #	4.5	2.17	1.70	1.25
Ce	14.7	12.2	N/A	N/A
Co	113	97.0	164	2.05
Cr *	1.38	1.25	N/A	N/A
Cs	14.0	14.2	25.5	1.13
Cu *	5.60	4.72	0.34	0.04
Er	1.24	1.22	N/A	N/A
Fe *	44.7	41.8	1.40	0.26
K #	6.78	6.75	1.11	1.54
La	10.4	9.26	N/A	N/A
Li	105	103	8.88	N/A
Mg *	557	564	151	108
Mn *	9.87	8.88	0.13	0.02
Mo	137	107	N/A	N/A
Na #	4.65	4.54	0.90	0.005
Nd	8.13	7.44	N/A	N/A
Ni	527	494	3.63	N/A
P #	2.12	1.72	1.42	1.06
Pb	N/A	N/A	20.5	N/A
Rb *	2.72	2.71	1.80	1.92
Sr *	1.58	1.27	1.52	0.53
U	9.07	6.15	N/A	N/A
V	445	421	N/A	N/A
Y	19.3	18.3	N/A	N/A
Zn *	39.0	36.7	5.26	4.28

* [mg/kg]. # [g/kg]. ^Indicates mineral present bellow detection limit (See Table S3). % Results are reported as mean ± standard deviation from three independent replicates.

Table 2. μ-CT instrument operational parameters.

**Micro-CT setting**	**Unit**	**Value**
Source Voltage	kV	40
Current	μA	250
Aluminum filter size	mm	0.5
Image pixel size	μm	17.45
Frame averaging	N/A *	3
Random movement adjustment	N/A	4
Rotation step	degree	0.2

* N/A = Not applicable.

Table 3. Detection limits for bone ICP-MS analysis.

Element	Detection limit [mg/kg]	Element	Detection limit [mg/kg]
Na	<40	Ag	<0.05
Mg	<44	Cd	<0.1
Al	<10	Sb	<0.04
P	<100	Cs	<0.02
K	<120	Ba	<0.04
Ca	<200	La	<0.01
V	<0.3	Ce	<0.02
Cr	<0.3	Pr	<0.01
Mn	<0.1	Nd	<0.02
Fe	<2	Sm	<0.04
Co	<0.04	Eu	<0.01
Ni	<0.1	Gd	<0.03
Cu	<0.2	Dy	<0.02
Zn	<20	Ho	<0.005
As	<0.1	Er	<0.01
Se	<0.2	Tm	<0.01
Rb	<0.04	Yb	<0.01
Sr	<0.04	Lu	<0.01
Y	<0.02	Pb	<0.1
Mo	<0.04	U	<0.02

Table 4. Parameters determined in cortical and trabecular bone using µ-CT analysis (Reproduced from Burrow, et al. [7]).

Characteristic	Abbreviation	Bone Type	Unit	Description	Desirability in relation to bone health
Bone volume	BV	Cortical	mm^3	Volume of the region identified as bone within the area assessed	↑ Desirable
Bone surface	BS		mm^2	Surface area of the region identified as bone within area assessed	
Bone surface to volume ratio	BS/BV		mm^2/mm^3	Ratio between the surface of the area identified as bone and the total volume of that area	
Bone surface density	BSD		mm^2/mm^3	Ratio between the region identified as bone to the total volume of the region assessed	
Number of pores	PN		number	Total number of all closed pores within the area assessed	↑ Undesirable
Volume of pores	PV		mm^3	Total volume of all open and closed pores within the area assessed	
Percentage porosity	P %		%	The volume of all closed pores as a percent of the total volume assessed	
Bone volume	BV/TV	Trabecular	%	Volume of the region identified as bone within the area assessed	↑ Desirable
Bone surface density	BS/TV		mm^2/mm^3	Ratio between the region identified as bone to the total volume of the region assessed	
Bone surface to volume ratio	BS/BV		mm^2/mm^3	Ratio between the surface of the area identified as bone and the total volume of that area	
Trabecular pattern factor (fragmentation index)	Tb.Pf		N/A*	An inverse measure of connectivity within the trabecular bone	↑ Undesirable
Trabecular thickness	Tb.Th		mm	Mean thickness of trabeculae	↑ Desirable
Trabecular separation	Tb.Ts		mm	Mean distance between trabeculae	
Trabecular number	Tb.N		1/mm	The average number of trabeculae in a given length of bone	
Degree of anisotropy	DA		N/A*	A measure of 3D symmetry from 1 to infinity with 1 meaning full isotropic (homogenous) characteristic and infinity full anisotropic characteristics	↑ Undesirable
Fractional Dimension	FD		N/A*	Indicator of surface complexity	↑ Desirable

* Unit-less measurement.
